# Images from a jointly-arousing collective ritual reveal affective polarization

**DOI:** 10.3389/fpsyg.2013.00960

**Published:** 2013-12-24

**Authors:** Joseph A. Bulbulia, Dimitris Xygalatas, Uffe Schjoedt, Sabela Fondevila, Chris G. Sibley, Ivana Konvalinka

**Affiliations:** ^1^Faculty of Humanities and Social Sciences, Victoria University of WellingtonWellington, New Zealand; ^2^LEVYNA Laboratory for the Experimental Research of Religion, Masaryk UniversityBrno, Czech Republic; ^3^Interacting Minds Center, Aarhus UniversityAarhus, Denmark; ^4^Center for Human Evolution and Behavior, Universidad Complutense de MadridMadrid, Spain; ^5^Department of Psychology, University of AucklandAuckland, New Zealand; ^6^Section for Cognitive Systems, Department of Applied Mathematics and Computer Science, Technical University of DenmarkLyngby, Denmark

**Keywords:** evolution, fire, Markov chain Monte Carlo, multi-level, religion, ritual

## Abstract

Collective rituals are biologically ancient and culturally pervasive, yet few studies have quantified their effects on participants. We assessed two plausible models from qualitative anthropology: **ritual empathy** predicts affective convergence among all ritual participants irrespective of ritual role; **rite-of-passage** predicts emotional differences, specifically that ritual initiates will express relatively negative valence when compared with non-initiates. To evaluate model predictions, images of participants in a Spanish fire-walking ritual were extracted from video footage and assessed by nine Spanish raters for arousal and valence. Consistent with **rite-of-passage** predictions, we found that arousal jointly increased for all participants but that valence differed by ritual role: fire-walkers exhibited increasingly positive arousal and increasingly negative valence when compared with passengers. This result offers the first quantified evidence for rite of passage dynamics *within* a highly arousing collective ritual. Methodologically, we show that surprisingly simple and non-invasive data structures (rated video images) may be combined with methods from evolutionary ecology (Bayesian Generalized Linear Mixed Effects models) to clarify poorly understood dimensions of the human condition.

## Introduction

**Figure 1:**
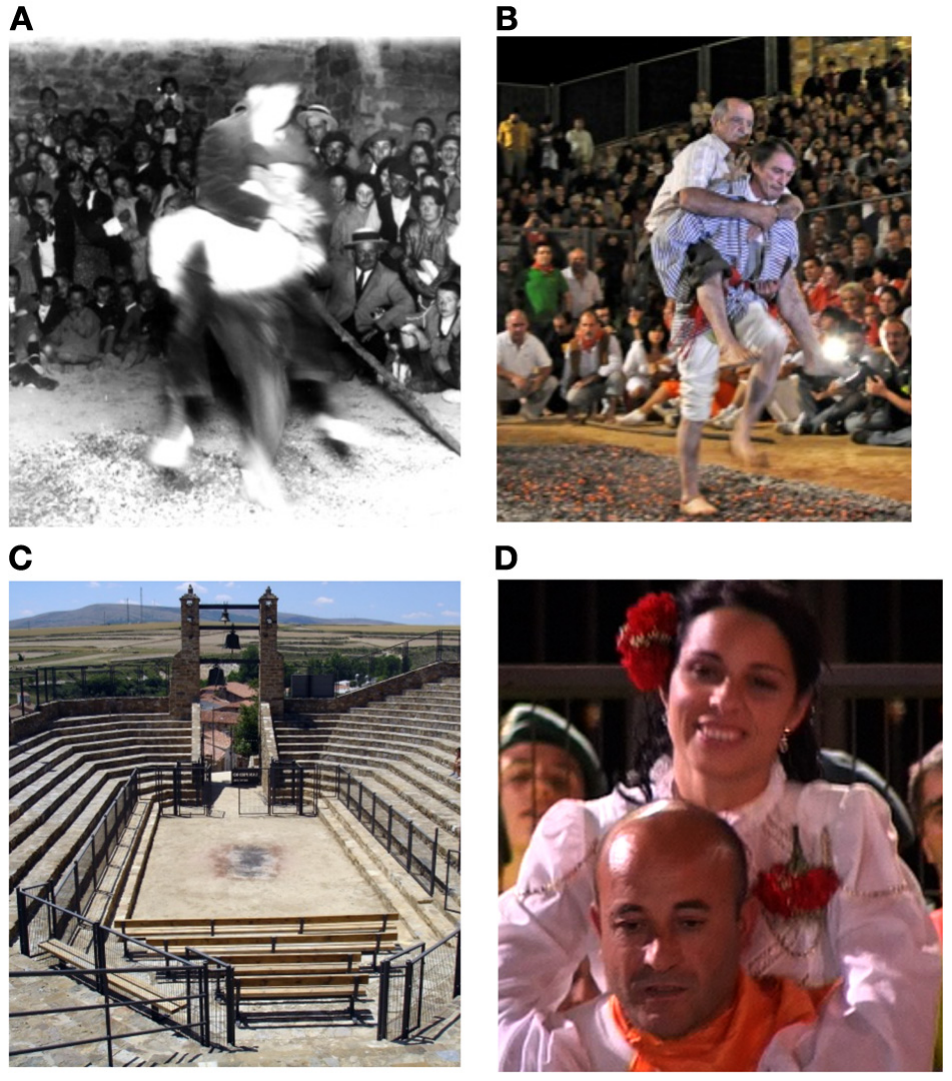
**Images from the San Pedro Fire-walking Ritual**. **(A)** is an image of a walker/passenger dyad from the early twentieth century. **(B)** is an image from a contemporary fire-walking ritual. Qualitatively, it would appear that there is a strong conservation of this ritual form. Such conservation is puzzling because the ritual involves pain, though without any obvious benefits to health or prosperity. Functionalists posit indirect benefits to prosperity from social bonding. **(C)** shows the purpose-built theater constructed for this ritual, which accommodates roughly 3000 spectators. Note the scorch marks on the pavement from previous fires. **(D)** shows a typical rated image from the middle stage of a fire-walk, hinting at different valence in the expressions of this fire-walker and passenger pair.

Collective rituals are symbolic activities lacking obvious biological interpretations because the goals of ritual action (causing it to rain, pleasing Zeus, obtaining knowledge of the future) are not obviously realized by the ritual actions themselves (dancing in circles, sacrificing livestock, reading animal entrails). Yet evidence for ritual activity extends to a time depth of over 150,000 years (White et al., 2003). What conserves such biologically puzzling behaviors? (Figures 1A,B).

It has been conjectured that collective rituals are conserved because they extend mutually enhancing cooperative commitments among unrelated partners (Irons, 1996; Rossano, 2006; Norenzayan and Shariff, 2008; Atran and Henrich, 2010; Gervais et al., 2011; Bulbulia, 2012). Recent studies have quantified enhancements to pro-sociality using historical evidence (Sosis and Bressler, 2003; Atkinson and Whitehouse, 2011) and by comparing responses between a variety of naturally occurring collective ritual practices (Sosis and Ruffle, 2003; Fischer et al., 2013; Xygalatas et al., 2013a). However, little is known about the proximate systems *within* rituals that drive post-ritual bonding. Two general models have been proposed.

1. **Empathetic sharing** conjectures that rituals increase perceived social unity by evoking shared emotional experiences culminating in an “ecstatic state of union” (Haidt, 2007, p. 1001; Haidt et al., 2008). In support of empathetic sharing, it has been found that collective rituals enhance feelings of oneness and increase charitable donations (Fischer et al., 2013; Xygalatas et al., 2013a). Notably, collective rituals have also been observed to coordinate the heart-rhythms of ritual participants (Konvalinka et al., 2011; Mueller and Lindenberger, 2011; Xygalatas et al., 2011; Vickhoff et al., 2013).
2. **Rite-of-passage** holds that post-ritual social bonding arises from structured ordeals, which appear to bring disproportionately high trauma to ritual initiates as compared with non-initiates (Turner, 1990; van Gennep, 2011/1961). It has been hypothesized that such ritual ordeals facilitate social bonding by channeling cognitive dissonance to social norms (Schjoedt et al., 2013) and by effecting lasting interpersonal memories among ritual cohort (Whitehouse, 2000). It has been conjectured that ritual evolved to both recruit and project signals of cooperative commitment (Bulbulia, 2004), and that ritual ordeals might function to verify one's commitment to a group (Sosis et al., 2007; Bulbulia and Sosis, 2011). Notably, recent studies have linked strong emotional expressions to the signaling of cooperative commitment (Schug et al., 2010).

Assessing these different models requires evaluating affective responses for different ritual roles *in situ*. To our knowledge, however, no previous study has systematically quantified within-ritual affective expressions. To this end, we applied Bayesian data analysis to rated images of participants in a highly-arousing traditional Spanish fire-walking ritual.

The fire-walking ritual we examined is ideally suited for evaluating the proposed theoretical models. There are two ritual roles in this collective ritual: fire-walkers and passengers. Physical ordeals vary by ritual roles. Fire-walkers must traverse a searing hot fire, bare-footed, in front of several thousand observers, carrying adult passengers on their backs. In accordance with **rite of passage**, one might expect differences in affective responses between those who walk the fire and those who are transported over the fire, feet unscathed. On the other hand, passengers of fire-walkers are always closely related family or friends. Studies from cognitive neuroscience indicate that people respond to the suffering of loved ones with evoked pain responses (de Vignemont and Singer, 2006). It is therefore conceivable that laying hold of a beloved fire-walker might elaborate expressions of joint suffering, in accordance with the predictions of **empathetic sharing**. A further advantage of investigating this fire-walking ritual comes from our quantification of heart rate synchronization in a previous study, which revealed a coupling in heart rate rhythms between fire-walkers and observers (Konvalinka et al., 2011). Importantly, levels of coupling were predicted by social relationships: close observers presented coupling and socially distant observers were uncoupled. This prior evidence for shared arousal at this fire-walking ritual enabled us to test our methods for detecting signals of affective responses from rated image data. Such a test is important for two reasons: it offers a chance to replicate the shared arousal finding using a different bio-marker, rated facial expressions. More fundamentally, corroborating evidence for shared arousal affords additional confidence for our method of assessing valence, the key affective dimension relevant to evaluating theoretical predictions (Figure 1D).

## Methods

### Ritual setting

The study was conducted in San Pedro Manrique Spain, a village of about 600 inhabitants situated in northeastern Spain. The fire-walking ritual is the culmination of the 8-day festival of San Juan, which occurs annually on June 23rd. Though evidence for the fire-walking ritual appears in the earliest town records dating to the late nineteenth century, its exact origins remain obscure. Comparable fire-walking rituals have been documented among Greek Christian communities (Anastenaria) since at least the medieval period (Xygalatas, 2013). During the mid 1970s, the village of San Pedro erected a purpose-built amphitheater to accommodate up to 3000 visitors to the event (see, Figure 1C), roughly five times the village's population.

The fire over which the walkers traversed was built from solid oak. It was about three meters long and one and a half meters wide. Our pyrometer reading indicated that the surface temperatures of the coal-bed on the day of our study reached 677°C prior to the fire-walking ritual. The fire's heat was palpable from the lower stands, roughly 7 m away. After arriving to the venue, brimming with cheering spectators, the fire-walkers performed a circular dance. They then took their position opposite the burning coals. A dissonant horn called each walker-passenger pair to their position, and fire-walks occurred in a series; each walk lasted about 4 s. There were 28 fire-walks during the year of our study (2008).

**Figure 2:**
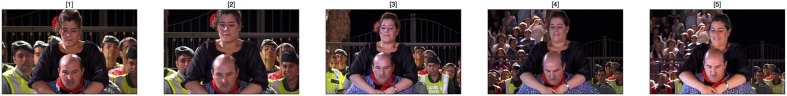
**Presentation of a typical sequence of rated images**. Raters were from the geo-cultural region in which the fire-walk took place, but had never attended. The sequence of images was presented in the order in which the ritual occurred. Raters were given a brief description of the ritual. We recruited raters from the region of the ritual and provided them with information about the ritual because we were interested in recovering contextually informed interpretations of affective responses. Said differently, we modeled culture as the natural context of affective interpretation rather than as a confound.

Images of walkers and passengers were extracted from high-definition video footage by a research assistant naive to our quasi-experimental hypotheses. The assistant was instructed to select the five clearest images for each fire-walker and each passenger from each of five phases of the fire walk: (1) entry, (2) early fire-walk, (3) middle fire-walk, (4) late fire-walk, and (5) completion. Images targeted the upper torso and facial regions of participants (see, Figure 2). In all, 42 participants from 26 fire-walks were assessed at five intervals of each fire-walk.

A total of nine raters from northern Spain unfamiliar with the ritual were recruited to rate images. As previous research has detected culturally specific norms in the expression of emotions (Matsumoto, 1989; Matsumoto et al., 2008), we used raters from the same geo-cultural region where the ritual took place (Appendix). No rater had previously attended this ritual. Raters were informed that the images were taken from a fire-walking ritual in San Pedro in Soria. Each rater was given a brief and general description of the ritual. We provided a context for the rating of images because we were interested in assessing the typical responses of a Northern Spanish audience to facial image data. We also presented video images in the order of the ritual event, again to recover a more ecologically realistic indicator for responses among people who inhabit this region. We instructed raters to judge emotional expressions along two affective dimensions: arousal and valence.

Ratings occurred on ordinal scales from 1 to 7. Combining all ratings of 42 participants yielded a total of 18,900 judgments—the units of analysis for our study. There were 1,112 cases in which raters judged images insufficiently clear to assess. Such judgments were recorded as missing and were imputed values during Markov chain Monte Carlo estimation (MCMC). Importantly, Bayesian mixed-effects regressions models simultaneously adjusted for the uncertainty resulting from such imputation to missing values. As indicated above, arousal ratings enabled us to assess our methods against previous evidence of coupled arousal during this same fire-walking ritual (Konvalinka et al., 2011). Valence ratings enabled us to evaluate theoretical predictions of the two models we considered, **ritual empathy** and **rite of passage**.

## Results

### Data exploration

**Figure 3:**
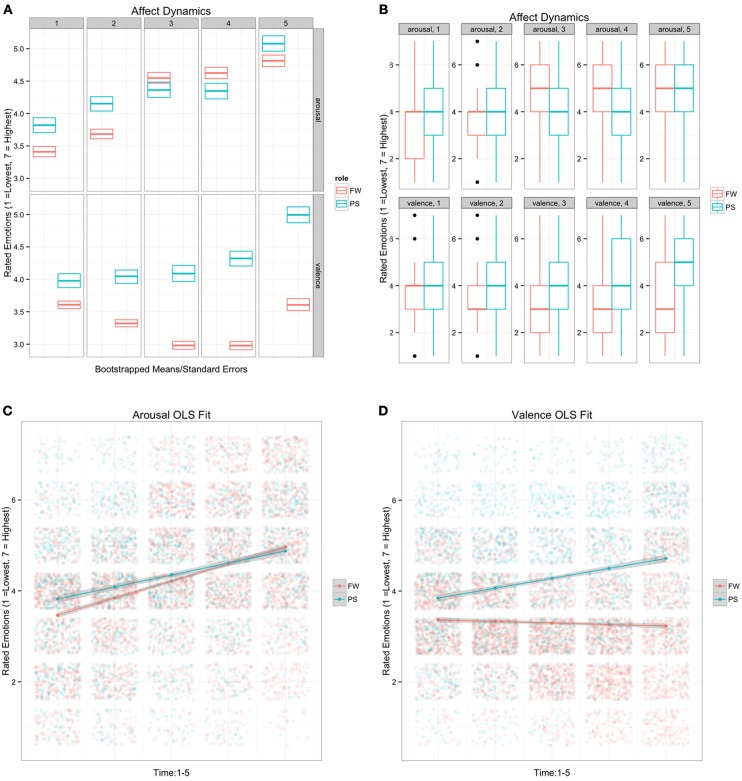
**Data presents exploratory graphs of the role-by-time trend using classical Ordinary Least Squares (OLS) estimation**. Panel **(A)** shows bootstrap means and standard errors from OLS regression, indicating statistically significant main effects for the interactions of role and time on both arousal and valence. Boxplots in Panel **(B)** and raw data plots with regression lines for **(C)** arousal and **(D)** valence suggest broad response variances among ratings of individual participant expressions at the different stages of the ritual. Doubt about the extent to which this diffuse pattern depends on rater effects must be included in any probability model. Dependencies from clustering parameters—time within individuals and events—must also be included within such a probability model because these dependencies invalidate the assumptions of classical regression and ANOVA. Bayesian Generalized Linear Mixed Effects models accommodate dependencies inherent in data such as ours while also propagating doubt arising from rater effects. Importantly, our interest was not in excluding a null hypothesis—our study has no null hypothesis—but rather in quantifying uncertainty about parameter estimates.

Data exploration suggested increasing arousal for each role over time (Figure 3). The pattern of arousal was broadly consistent with observations of synchronized arousal from our previous study, which used heart rate analysis to quantify joint arousal during this same event (Konvalinka et al., 2011). Tables 1, 2 show “statistically significant” main effects and interactions for role and valence. Raw data plots offer little indication of ritual empathy along the valence dimension. Rather, passengers exhibit a positive valence trend whereas fire-walkers exhibit a somewhat negative valence trend. Boxplots hint at fire-walker recovery during the final phase. Importantly, enthusiasm from “statistically significant” *p*-values is unjustified: any formal model for affective responses must adjust for clustering effects within/across the rituals and for uncertainties owing to rater effects. We used Bayesian GLMMs because they allowed explicit modeling of clustering co/variances and rater effects, and because they allowed us to quantify uncertainty based on our prior belief that subjective ratings of affective responses would be variable.

**Table 1:** **Coefficients for OLS regression for Arousal outcomes**.

**Arousal**	**Estimate**	**Std. error**	***t* value**	**Pr(>|t|)**
(Intercept)	3.09206	0.04539	68.125	<2e-16^***^
rolePS	0.46512	0.07513	6.191	6.24e-10^***^
Time	0.37453	0.01365	27.436	<2e-16^***^
rolePS:time	−0.11027	0.02344	−4.703	2.60e-06^***^

*** *p < 0.001*.

**Table 2:** **Coefficients for OLS regression for Valence outcomes**.

**Valence**	**Estimate**	**Std. error**	***t* value**	**Pr(>|t|)**
Intercept	3.39960	0.04073	83.469	<2e-16^*^
rolePS	0.22773	0.06745	3.376	0.000738^*^
time	−0.03392	0.01226	−2.768	0.005657^**^
rolePS:time	0.25173	0.02104	11.963	<2e-16 ^*^

^***^p < 0.001;

** p < 0.01;

* *p < 0.05*.

### Rater reliability: intraclass correlation

An intraclass correlation coefficient (ICC) describes the expected correlation between outcomes of a particular group member (level of a random effect), averaging over one or more of the other random effects (the repeatably). We calculated an intraclass correlation on the raw data scale for the average agreement and consistency in ratings. The two-way ICC(2,9) for average agreement was 0.79, *F*_(898, 112)_ = 6.16, *p* ≤ 0.0001, CI(0.72, 0.83) and the two-way ICC(2,9) for average consistency was 0.84, *F*_(909, 7272)_ = 6.43, *p* ≤ 0.0001, CI(0.83, 0.85). We used the ICC 2,k from our interest in the repeatability of rater effects.

### MCMC models

**Figure 4:**
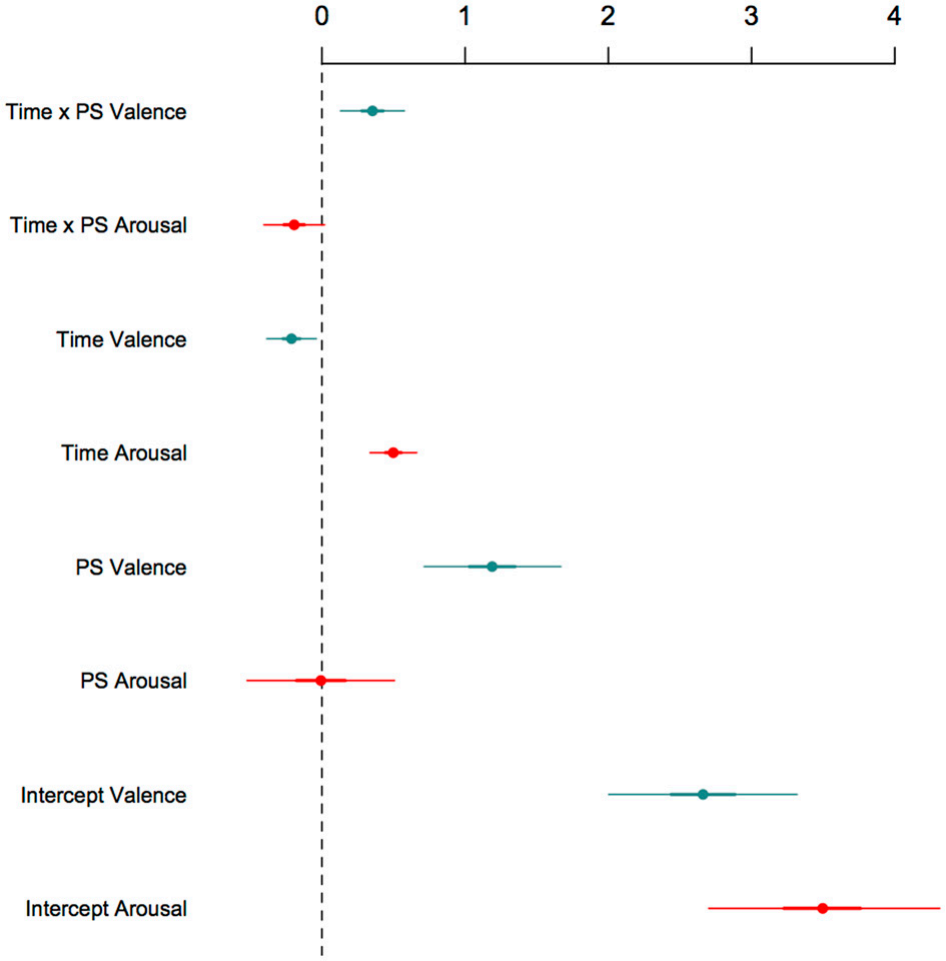
**This figure shows regression coefficients for effects modeled as fixed**. Estimates are given on the latent scale. To recover coefficients on the data scale, add 1. Time effects were centered at 0. We coded fire-walkers as 0 and passengers as 1. Hence, the intercepts denote the expected arousal and valence for fire-walkers at the middle point of the ritual (on the latent scale). Our interest was in the role × time interaction, which shows a pattern of diverging mean valence for role over time. As indicated here, fire-walkers were found to be increasingly negative in their valence whereas passengers were found to be increasingly positive. These findings are consistent with the predictions of the rite-of-passage model.

Model selection was guided by the Deviance Information Criterion (Speigelhalter et al., 2003). We found that for the model including the role × time interaction, the DIC score for the model including the role × time interaction was DIC_role × time_ = 48,819. This substantially improved on the DIC score of the empty model, DIC_intercept only_ = 57,033 (Δ DIC = 8214) and also improved on the DIC score of a model that excluded ritual role effects, DIC_time_ = 48,854 (Δ DIC = 54). We tested a quadratic model for the role × time interaction, however, we found the quadratic model did not improve the overall model fit of the linear model as measured by the DIC, DIC_role × time^2^_ = 48,880 (Δ DIC = 61). We therefore report the linear model as the best performing model. However, we advise caution about rejecting the quadratic model, given its DIC is relatively close to the preferred model. Notably, both the linear and quadratic models yield identical inferences: both support **rite-of-passage**. Therefore, a preference does not affect the theoretical conclusions of this study. All models included co/variance components for the following effects modeled as random: the intercepts and slopes of individuals over time (time here assessed as a factor), for events co/variances (a factor with 26 levels), and raters co/variances (a factor with nine levels) (see Appendix). The posterior distributions for effects conditionally modeled as fixed are given in Table 3 and are graphed in Figure 4.

**Table 3:** **Locations for effects modeled as fixed in the MCMC analysis, along with the probabilities of sign errors (type_S_) and magnitude errors (type_M_)**.

**Location**	**Posterior mean**	**l-95% CI**	**u-95% CI**	**Eff. samp**	**pMCMC**	**type**_**S**	**type**_**M**
Intercept arousal	3.496693	2.699159	4.313096	7526	<1e-04^*^	0.0000	0.0001
Intercept valence	2.661037	2.008884	3.324146	8007	<1e-04^*^	0.0000	0.0002
Main arousal:rolePS	−0.007176	−0.504739	0.515628	5013	0.9742	0.4871	0.0063
Main valence:rolePS	1.188421	0.737236	1.685335	7468	<1e-04^*^	0.0000	0.0083
Main arousal:time	0.498418	0.340313	0.664830	6346	<1e-04^*^	0.0000	0.0017
Main valence:time	−0.212262	−0.382479	−0.037495	5963	0.0162^*^	0.0081	0.1033
arousal:rolePS X time	−0.193590	−0.405325	0.018015	6254	0.0734^*^	0.0367	0.1438
valence:rolePS X time	0.353192	0.130967	0.577182	5911	0.0030^**^	0.0015	0.0613

^***^pMCMC < 0.001;

** pMCMC < 0.01;

* *pMCMC < 0.05*.

Consistent with past studies showing synchronous arousal for this fire-walking ritual, we did not find overall differences in average arousal for the different roles, *b*_arousal:rolePS_ = −0.007, 95% Credible Interval (HPD) from −0.51 to 0.52. Ignoring role, we found that average arousal increased over time for all participants, *b*_arousal:time_ = 0.50, 95% Credible Interval (HPD) from 0.34 to 0.66. Ignoring time, we found that overall valence differed by role, with passengers on average exhibiting more positive responses, *b*_valence:rolePS_ = 1.19, 95% Credible Interval (HPD) from 0.74 to 1.69, though ignoring role, overall average valence was found to be slightly lower over time, *b*_valence:time_ = −0.21, 95% Credible Interval (HPD) from −0.38 to −0.03.

Our quasi-experimental interest centered on the interaction of the role × time treatments. We found that arousal responses were similar across the different roles *b*_arousal: role × time_ = −0.19, 95% Credible Interval (HPD) from −0.41 to 0.02. Recalling that in Bayesian regression, parameter estimates may be directly interpreted on the probability scale, the fact that the posterior mean estimate for the role × arousal effect is both close to zero, and crosses zero at its 95% HPD, suggests that passengers and fire-walkers were similarly (increasingly) aroused over time. By contrast, the expected average valence among passengers was found to be relatively positive when compared to the valence of fire-walkers *b*_valence:role × time_ = 0.35, 95% Credible Interval (HPD) from 0.13 to 0.58. Hence, we infer valence effects over time were reliably opposed depending on ritual role: fire-walkers exhibit lower average valence over time compared with passengers who exhibit positive average valence over time.

To facilitate interpretation of our results, we calculated the expected average responses at the posterior means of the coefficient estimates for different stages of the fire-walk depending on ritual role. Recalling that rating scales were ordinal ranging from 1 (minimum) to 7 (maximum), the expected average arousal in passengers at the start of the ritual is $\tilde{\text{arl}}$_p:start_ = 3.88, at the middle: $\tilde{\text{arl}}$_p:mid_ = 4.49, and at the end: $\tilde{\text{arl}}$_p:end_ = 5.01. The expected average fire-walker arousal at the start is $\tilde{\text{arl}}$_fw:start_ = 3.50, at the middle: $\tilde{\text{arl}}$_fw:mid_ = 4.51, and at the end: $\tilde{\text{arl}}$_fw:end_ = 5.51. These estimates reflect a pattern of jointly increasing average arousal for both ritual roles.

Turning to valence, the expected average valence among passengers at the start of the ritual is $\tilde{\text{val}}$_p:start_ = 4.57 at the middle: $\tilde{\text{val}}$_p:mid_ = 4.85, and at the end: $\tilde{\text{val}}$_p:end_ = 5.13. By contrast, the expected average valence among fire-walkers at the start is $\tilde{\text{val}}$_fw:start_ = 4.09, at the middle: $\tilde{\text{val}}$_fw:mid_ = 3.66, and at the end: $\tilde{\text{val}}$_fw:end_ = 3.24.

Notably, these expected averages are consistent with **rite-of-passage** predictions: fire-walkers increasingly suffered across their trials by fire, whereas passengers presented increasing delight. Though passengers were socially close to fire-walkers, and indeed physically embraced fire-walkers across the fire, we found that average valence responses over time among fire-walkers and passengers consistently *diverged*. Put simply, we find little evidence of empathetic response in rated valence expressions among fire-walkers and passengers.

A virtue of Bayesian estimation is its power to recover signals of interest from noisy indicators by propagating across the model, the uncertainty that noise components introduce. We can compare estimates of noise introduced from rater co/variances to the co/variances of individual responses. We estimated the random variance calculated for individual valence intercepts in the final stage of the fire-walk as var(time:5)^*v*^ = 2.68, 95% Credible Interval (HPD) from 1.49 to 4.13. This variance is roughly three times greater than that of rater's effects, *var*(raters)^*v*^ = 0.59, 95% Credible Interval (HPD) from 0.18 to 1.34. We estimated the arousal variances for raters as *var*(raters)^*a*^ = 0.96, 95% Credible Interval (HPD) from 0.30 to 2.1, indicating greater variance in the ratings of arousal when compared to valence. However, these variance components in ratings of arousal are of a similar magnitude to the variance of individual arousal at four of the five stages of the fire walk. We estimated all other variance and co-variances as less than 1. We estimated the residual units co-variance for arousal as *cov*(units)^*a*/*v*^ = 0.36, 95% Credible Interval (HPD) from 0.31 to 0.40. This indicates that expected arousal and valence are positively correlated. Higher valence was associated with higher arousal.

## Discussion

Though collective rituals are human universals, within-ritual social-affective cognition remains poorly understood. We used rated images of participants faces/upper torsos to evaluate the predictions of two theoretically debated models for within-ritual social-affective cognition: **ritual empathy**, which predicts affective *merging* irrespective of ritual role and **rite-of-passage** which predicts affective *diverging*, with ritual initiates presenting more negative valence in their expressions. To handle noisy, correlated, and dependent data structures resulting from rated images extracted from raw video footage, we adapted statistical methods from evolutionary ecology (Bayesian multi-level regression). Consistent with previous results (Konvalinka et al., 2011), and supporting our method for affective analysis, we found that average arousal jointly increased over time among all participants, but more steeply among fire-walkers. Relevant to our experimental models, we found that average valence predictably differed by ritual role, and crucially, in opposing directions: fire-walker valence became increasingly negative on average compared to increasingly positive valence among passengers. These results offer preliminary support for the **rite-of-passage** model. For the different roles, we found that arousal *merged* but valence *diverged*.

Enthusiasm for the **rite-of-passage** model, however, must be balanced with an appreciation of our small sample size and of the cultural specificity of this ritual. Clearly more studies investigating high arousal rituals are needed. Confining attention to our results, it has been conjectured that the suppression of emotions forms part of a ritual ordeal (Schjoedt et al., 2013). We think it is possible that fire-walkers suffered more than they let on. Other theorists attribute trance-like states to ritual-initiates which are hypothesized to buffer initiates from negative sensations (Ward, 1984). Perhaps raters over-attributed negative affect to fire-walkers because raters expected negative affect. (Yet we again emphasize that a previous study found remarkably high levels of arousal in fire-walker heart-rates during their ordeals, indicating a strong affective response as operationalized by the heart-rhythm bio-marker.) How to characterize affective responses in highly arousing collective rituals remains unclear: we hope that future research will improve on the rough indicators we used in this study. As for passengers, the absence of evidence for shared valence does not rule out empathetic responses. Perhaps passengers suffered on the inside in ways we did not detect. Our model cannot rule out such possibilities, and we caution against over-zealous inferences.

Nevertheless, we also find it credible that empathy failed in this ritual. Such failures are, after all, qualitatively familiar human experiences. Consider the dissertation defense. At this academic trial by fire, the inquisitor's glee is the defendant's terror. Later, however, inquisitor and initiate may greet as colleagues. By appearances, if solidarity is forged from such an ordeal, is not straightforwardly from a sharing of empathetic experience. In the San Pedro ritual, it is possible that empathy failed because passengers did not see the faces of fire-walkers, and as performers in the ritual themselves, passengers may have been focusing more on themselves, rather than on fire-walker feelings. Of course, the same could be said of fire-walkers who failed to connect with the increasing elation of their passengers (!) Whether mechanisms of special ordeal and predictably different emotions conspire to amplify post-ritual solidarity is an important horizon for future research. We hope our study will spark further empirical interest in the complex and multi-faceted social and affective processes that underpin such old, undying collective practices.

To summarize, our study makes an important contribution both for its methodology and for its findings. On the methodology front, we illustrate how non-invasive methods for data capture (video) may be combined with statistically appropriate models from evolutionary ecology (Bayesian GLMMs) to clarify puzzling and hard-to-access dimensions of the human condition which have long eluded qualitative anthropologists. Put simply, we show how tools from the biological sciences may be adapted to address questions about human social cognition in natural human ecologies. As for our results, we offer the first quantitative evidence in support of a longstanding **rite of passage** model for ritual experience. The focus of much current ritual research centers on the manner by which rituals evoke shared experiences (Haidt, 2007, p. 1001). Such a focus is sensible, given evidence of *post*-ritual bonding. However, the **rite-of-passage** theory, an old and mainly overlooked chestnut from qualitative anthropology, proposes that *within*-ritual differences in affective experiences fuel post-ritual solidarity. In accordance with **rite-of-passage** predictions, we find that average valence trends in opposite directions at a Spanish Fire-walking ritual depending on whether one is a fire-walker or a passenger. The prospect that rituals might build communities by disproportionately traumatizing some participants, but not others, is practically important to the larger project of understanding ritual solidarity. Though rituals are strong instigators of collective action, evoked empathy *within* a ritual need not be the universal affective tether by which collective rituals bind people together.

### Conflict of interest statement

The authors declare that the research was conducted in the absence of any commercial or financial relationships that could be construed as a potential conflict of interest.

## Appendix

### Modeling rated images

Prior to this study, we solicited advice from Paul Ekman, a leading authority on estimating emotions from facial images, about how to best assess ritual emotions from video data. Ekman advised that we obtain an unedited continuous sample of 5 min hi-resolution video for each participant, “lit well enough to see when the lower eyelid is tensed” (Ekman, personal communication) This advice is in accordance with Eckman's manual (Ekman and Rosenberg, 1997). We were grateful for Ekman's help, however, we soon discovered that the natural human ecology of a Spanish fire-walking ritual posed insurmountable challenges to following it. In our study, each fire-walk lasted less than 5 s. Moreover, we had limited control over aspect orientation and almost no control over lighting. Two fire-walking events were omitted not from carelessness but rather because cheering spectators entirely obstructed the camera. Resulting image data were generally clear enough to detect facial/upper torso expressions but invariably of a lower quality than what would be typical of an experimentally controlled design. It is for this reason that we turned to methods from evolutionary ecology where models have been developed to handle imperfect data structures. Linear mixed effects regression enabled us to handle correlations in the data which must be accounted for because they invalidate the standard assumptions of ANOVA, leading to downwardly biased estimates of standard errors (Zuur, 2009). An ordered multinomial probit link function enabled us to model latent outcomes from observed ratings as drawing from a truncated multivariate normal distribution (see below, though note that assuming a guassian distribution resulted in practically equivalent theoretical inferences). Finally, Bayesian estimation allowed us to propagate uncertainty arising from differences in rater judgments across our regression model so that we could use all rater judgments without averaging or attempting to coerce *post hoc* agreements among raters. We adopted our Bayesian estimation method for handling rater responses directly from (Adams et al., 2012), and we recommend wider adoption of the Adams et al. model, bearing in mind that all methods must be adjusted to whatever theoretical task is to hand. For general introductions to Bayesian Data Analysis, see: (Gelman et al., 2004; Lynch, 2007; Kruschke et al., 2012).

Beyond the realities of capturing data from the field, we had to make decisions about how to present images to raters, how many raters to use, and what to tell them. How one should code affective responses depends on the theoretical ambitions of one's study, and we strongly resist the idea of canned solutions. During the past several decades, careful study of the invariant properties of emotional displays in humans have given rise to categorical coding schemes of affective signals (Ekman, 1999). These schemes identify emotional states by attending to activations in facial musculature (Ekman, 1975). The categorical paradigm has developed techniques for evaluating the honesty of facial expressions from subtle, secondary signals, which experts may detect (Ekman and Rosenberg, 1997). Such categorical coding schemes have been developed to help understand correlations between affective displays and affective states (Ekman and Rosenberg, 1997). Such an analysis has its strengths, but it also has its limitations. Ekman and Friesen observe that facial expressions are governed by culturally variable norms. Norms for the expression of fear, for example, vary by context (Ekman and Friesen, 1977). Inferences from fear signals to the psychological states that underpin their expression demand a local knowledge of cultural display rules (Heine et al., 2001; Matsumoto et al., 2008; Matsumoto and Hwang, 2010). Matsumoto generalizes from fear, suggesting that many “biological” (by which he means genetic) and “cultural” properties' of emotion differ (Matsumoto and Juang, 2013). Importantly, culture also influences the perception of emotion, not merely its display (Matsumoto, 1989). Such variation extends from broader cultural settings to narrow communicative contexts (Fernandez-Dols et al., 2002). Individual-level variation in the expression of emotion, moreover, has also been observed (Larsen et al., 1986; Koole, 2009; Matsumoto and Hwang, 2010) and some cortical control mechanisms that are involved in regulating the expression of emotional expressions have been mapped (Ochsner and Gross, 2005). Adding to such complexity, researchers disagree about the level of analysis appropriate for studying emotional expressions (Scherer and Ellgring, 2007). It is even debated whether the determinants of emotional displays should be modeled by categorical or continuous variables (Barrett, 1998; Gunes and Schuller, 2012), and indeed even more fundamentally, whether the very notion of an emotion independent of *some* interpretation makes any biological sense (Darwin, 1872; Griffiths, 1997) or any psychological sense (James, 1884; Barrett et al., 2009).

We cannot evaluate such philosophical debates with our study. Based on evidence of culturally specific display rules, we assessed arousal and valence using untrained, but culturally appropriate observers, hoping for enough average clarity in rater signals to identify trends in expressions for the different ritual roles. The two affective dimensions carry a long history of reasonable validation (Watson et al., 1999). Strong activations in these dimensions also play a role in memory suppression (Marx et al., 2008), which we observed in another study of this ritual (Xygalatas et al., 2013b). More fundamentally, arousal dynamics were relevant to checking our methods against past findings of joint-arousal and valence was relevant to assessing the predictions of the two theoretical models at hand, **ritual empathy** which predicts converging valence and **rite of passage** which predicts diverging valence. We note that such dimensions hardly exhaust the emotional complexity of a fire-walking ritual. We therefore emphasize once again the need for future research to improve on the coarse-grained theoretical constructs we used in this study.

### Construction of linear predictor

To model expected affective responses, we constructed multivariate Generalized Linear Mixed-Effects Models using the MCMCglmm package (Hadfield, 2010) in R (R Core Team, 2012). The model equation for our multivariate generalized linear model can be written in matrix notation:

$$\begin{array}{l} \;y^{*}\sim multinom\left(\eta ,\;\left(\begin{array}{cc} 1 & \sigma^{val}\\ \sigma^{aro} & 1 \end{array}\right)\right)\\ \eta =X_{\alpha }^{*}\alpha +X_{\rho }^{*}\rho +X_{\tau }^{*}\tau +X_{\rho \tau }^{*}\kappa +Z_{\text{at}}^{*}i+Z_{\text{e}}^{*}e+Z_{\text{r}}^{*}r \end{array}$$

- superscript ^*^ denotes the joint outcomes of arousal and valence (stacked)
- **y**^*^ denotes a (stacked) column vector for the latent arousal and valence variables
- the coefficient vector α locates the intercepts for the expected outcome of arousal and valence at the middle stage of the event (time is centered) for fire-walkers (the baseline) when all other predictors are set to zero
- the coefficient vector ρ locates the main effects of time on arousal and valence
- the coefficient vector τ locates the main effects of role on arousal and valence
- the coefficient vector κ locates the interaction of role × time on arousal and valence
- a co/variance components vector **i** adjusts for the co/variances of arousal and valence at random intercepts and slopes within individuals across the five intervals
- a variance components vector **e** adjusts for the co/variances of arousal and valence within the 26 events
- a variance components vector **r** adjusts for the co/variances of arousal and valence among the nine raters
- residual variances are fixed to 1 because variances components are not defined in an ordered multinomial model, however, residual covariances are estimated.

Outcomes were passed to a categorical probit link function, which estimated latent variables for the **y**^*^ outcome column vector. Jarrod Hadfiedld, creator of MCMCglmm, reports that for this package the probability of switching from one latent outcome threshold or cut-point to another on a latent scale of c-1 cut-points is:

$$\mathrm{Pr}(y=c)=\Phi \left(\gamma_{c}|\eta ,\;1\right)-\Phi \left(\gamma_{c-1}|\eta ,\;1\right)$$

- η is the model, γ_*c*_ is a cut-point and Φ is the probit function with the total probability of c summing to 1 (guaranteeing a probability for each measure) (Hadfield, 2010).

### Bayesian estimation

In Bayesian regression, there is little distinction between fixed and random effects (Gelman and Hill, 2007; Hadfield, 2012), however, the convention helps to identify model features. Priors for the fixed components of the model were non-informative, assuming a mean of zero and variance of 10^8^. We used parameter expanded priors (Hadfield, 2010, p. 128) as recommended by Gelman (2006), however use of inverse-Wishart priors for the random effects variances made no practical difference to theoretical inferences, as the posterior distribution of parameter co/variances were very similar. Put simply most of the information from the McMC estimation came from information in the data, not from the priors we assigned. Residual variances are not identified in a multinomial probit model so were fixed to one (Hadfield and Nakagawa, 2010). Residual co-variances were estimated assuming a flat prior, as Hadfield advises for ~cor(units) covariance matrix (Hadfield, 2012, p. 73). The indicator variables for time were centered to improve mixing, making the intercept interpretable as the expected outcome at the start of the ritual when all predictors are set to set to zero. Note: fire-walkers were coded as zero and passengers were coded as 1. The default number of cycles in MCMCglmm is 13,000. We allowed models to run in MCMCglmm for 110,000 cycles, with a thin interval of 10 (the default setting). The first 10,000 posterior draws were discarded. MCMC chains mixed well and there was no appreciable autocorrelation detected (all chains showed autocorrelation less than 0.05, using the MCMCglmm autocorr function). All chains were plotted using the MCMCglmm plot function. The MCMCglmm call for best performing model is written
